# Early 2 factor (E2F) transcription factors contribute to malignant progression and have clinical prognostic value in lower-grade glioma

**DOI:** 10.1080/21655979.2021.1985340

**Published:** 2021-10-07

**Authors:** Haitao Luo, Chuming Tao, Xiaoyan Long, Xingen Zhu, Kai Huang

**Affiliations:** a Department of Neurosurgery, The Second Affiliated Hospital of Nanchang University, Nanchang, Jiangxi Province, China; b Scientific Research Center, East China Institute of Digital Medical Engineering, Shangrao, Jiangxi Province, China; c Institute of Neuroscience, Nanchang University, Nanchang, Jiangxi Province, China

**Keywords:** E2F transcription factors, lower-grade glioma, risk model, overall survival, gene expression profile

## Abstract

Early 2 factor (E2F) genes encoding a family of transcription factors are significantly associated with apoptosis, metabolism, and angiogenesis in several tumor types. However, the biological functions of E2F transcription factors (E2Fs) and their potential involvement in the malignancy of lower-grade glioma (LGG) remain unclear. We explored the effects of the expression of eight E2F family members on the clinical characteristics of LGG based on the Chinese Glioma Genome Atlas (CGGA), The Cancer Genome Atlas (TCGA), and GSE16011 datasets. Two LGG subgroups were identified according to the consensus clustering of the eight E2Fs. We employed the least absolute shrinkage and selection operator (LASSO) Cox regression algorithm for further functional experiments and the development of a potential risk score. Two categories of patients with LGG were identified based on the median risk scores. We then developed a nomogram based on the results of the multivariate analysis. Real-time quantitative polymerase chain reaction (RT-qPCR) and immunohistochemistry were performed to validate the bioinformatics results. Our results indicated that E2F family members were significantly involved in the malignancy of LGG and might serve as effective prognostic biomarkers of the disease.

## Introduction

The early 2 factor (E2F) family includes eight critical members (E2F1, E2F2, E2F3, E2F4, E2F5, E2F6, E2F7, and E2F8), which share highly similar DNA-binding domains that interact directly with consensus sequences [1,2]. Members of the E2F family participate significantly in the oscillatory nature of the cell cycle by forming a core transcriptional axis [3,4]. The E2F transcription factors (E2Fs) also exert biological effects on pathways other than cell cycle regulation to contribute to malignant progression, such as apoptosis, angiogenesis, and metabolism [5–8].

The eight E2F family members were classified into three subgroups according to sequence homology and activity (activator protein, atypical repressor, and canonical repressor). Each member displays different expression and functional patterns consistent with their sub-categories [9,10]. The expression of activator proteins (E2F1-3) peaks in the G1-S phase, while atypical repressors (E2F7-8) show increased expression in the late S phase and canonical repressors (E2F4-6) are constitutively expressed during all phases [9]. Several protein complexes mediate the ability of E2Fs to bind DNA and regulate transcription [10]. E2Fs are largely self-regulated, and their subcellular localization is dependent on several factors. Moreover, multiple modifications of E2Fs have been identified that affect their expression, function, stability, and location [11–13].

Accumulating evidence strongly suggests that high expression of E2F family members is significantly associated with malignant progression in several tumor types [14–18]. For example, a strong positive correlation between E2F over-expression and poorer overall survival (OS) in patients with pancreatic tumors has been reported [14]. E2F1 and E2F2 expression levels were also significantly associated with a pro-angiogenic gene in breast cancer, which potentially conferred a more invasive phenotype [15]. E2F8 was significantly upregulated in lung cancer compared to normal lung tissue and was essential for cancer cell maturation [16,17]. Furthermore, E2F1 down-regulation suppressed tumor growth and invasion in bladder cancer [18]. These findings suggest that E2Fs contribute to the malignant progression of tumors.

While the tumor-promoting roles of E2Fs in multiple cancer types have been reported, limited knowledge exists on their biological functions and potential involvement in the malignancy of lower-grade glioma (LGG). Thus, we systematically investigated the relationship between E2F family members and LGG malignancy based on the clinicopathological characteristics of patients. Patients with LGG in the Chinese Glioma Genome Atlas (CGGA) dataset were sorted into two clusters, cluster1 and cluster2, by consensus clustering analysis based on E2Fs expression. The cluster1/2 subgroups affected the prognosis and clinical characteristics of LGG and showed strong associations with many critical biological processes, signaling pathways, and hallmarks of malignant LGG. We further selected four E2Fs using least absolute shrinkage and selection operator (LASSO) Cox regression analysis to derive risk scores, which facilitated the sorting of patients in both training (CGGA) and validation (The Cancer Genome Atlas (TCGA) and GSE16011) datasets into two categories. Finally, real-time quantitative polymerase chain reaction (RT-qPCR) and immunohistochemistry assays were performed to validate the bioinformatics results. Our collective results implicated the involvement of the eight E2Fs in malignant progression and might serve as prognostic biomarkers for LGG.

## Materials and methods

### Data collection

The RNA-seq transcriptome data and clinicopathological data of LGG samples were obtained from the CGGA (n = 443) (https://www.cgga.org.cn/), TGGA (n = 462) (https://portal.gdc.cancer.gov/), and GSE16011 datasets (n = 109) (https://www.ncbi.nlm.nih.gov/geo/query/acc.cgi?acc=GSE16011). The clinicopathological information is presented in Supplementary Table 1. Samples with incomplete data on survival time and survival status were excluded from the survival analysis. We normalized the RNA-seq data obtained from the CGGA, TCGA, and GSE16011 datasets.

### Selection of E2F family members

We selected eight E2F genes based on published literature [1,2] and mRNA expression data acquired from the CGGA, TCGA, and GSE16011 datasets. Next, we compared the translation levels of the E2Fs in brain and central nervous system (CNS) cancer samples to those of the corresponding normal control samples based on the ONCOMINE dataset (https://www.oncomine.org/) [19]. We also explored the mutation rates of the eight E2Fs and investigated their relationships with tumor immunology.

### Bioinformatic analysis

To explore the potential functions of the eight E2Fs in LGG, the R package ‘limma’ was applied to determine the associations between E2Fs expression patterns and the clinical characteristics of samples from the CGGA, TCGA, and GSE16011 datasets [20]. The relationships between the eight E2F genes were examined via Spearman’s correlation tests. A protein-protein interaction (PPI) network of the eight E2Fs was constructed by the STRING database and Cytoscape software [21]. Next, 443 patients with LGG from the CGGA dataset were sorted into two groups using the R package ‘ConsensusClusterPlus’ [22]. The grouping results were further validated using principal component analysis (PCA). Kaplan-Meier curves were plotted to analyze the OS of patients with LGG [23]. The R packages ‘clusterProfiler’, ‘enrichplot’, and ‘ggplot2’ were applied to perform Gene Ontology (GO) and Kyoto Encyclopedia of Genes and Genomes (KEGG) pathway analyses of genes showing differential expression between cluster1 and cluster2 [24]. Gene set enrichment analysis (GSEA) was then conducted to validate the functions of these differentially expressed genes.

Univariate Cox regression analysis was performed to determine the prognostic value of the E2F genes. We employed LASSO Cox regression algorithm for further functional experiments and the development of a potential risk score [25]. The minimum criteria were utilized to define four genes and select the optimal penalization coefficient lambda. We calculated the risk scores for all patients in the CGGA, TCGA, and GSE16011 datasets using the following formula: risk score = [E2F4 expression * (0.1612)] + [E2F7 expression * (0.3341)] + [E2F3 expression * (0.0687)] + [E2F2 expression * (0.3455)], in which we determined the coefficients by the minimum criteria and the transformed relative expression value of each selected E2Fs could be found in the datasets. Two categories of patients with LGG were identified based on the median risk scores. We employed a time-dependent receiver operating characteristic (ROC) curve to evaluate the prediction efficiency of our prognostic risk model [26]. We also developed a nomogram based on the results of multivariate analysis using the R packages ‘rms’ and ‘foreign’. The nomogram performance was evaluated by concordance index (C-index) and by comparing nomogram-predicted versus observed Kaplan-Meier estimates of survival probability [27].

### Use of RT-qPCR and immunohistochemistry assay to validate bioinformatics results

Normal brain tissues (NBT) and LGG tissues of patients were obtained from the Department of Neurosurgery of the Second Affiliated Hospital of Nanchang University between January 2017 and January 2021. Total RNA was isolated from the frozen tissue specimens using TRIzol reagent (Invitrogen, Carlsbad, CA, USA) following the manufacturer’s instructions. RT-qPCR was performed using a LightCycler® 480 Real-Time PCR System. Four selected E2Fs (E2F7, E2F4, E2F3, and E2F2) were assayed by qPCR on an Applied Biosystems real-time instrument using three-step amplification. The gene expression levels were measured using the comparative cycle threshold (ΔΔCt) method. The sequences of the forward and reverse primers for the four E2Fs family members are listed in Supplementary Table 2. All samples were repeated in triplicate. We also performed an immunohistochemistry assay on human tissues as previously described [28].

### Statistical analysis

Wilcoxon tests were applied to compare the risk scores and eight E2Fs between pairs of subtypes with the different clinical characteristics in LGG. Univariate and multivariate Cox regression analyses were used to determine the independent prognostic value of the risk scores and the nomogram, and Kaplan-Meier survival curves were generated to compare the OS of patients with LGG between different categories. Statistical analyses were conducted using SPSS for Windows, version 16.0 (SPSS Inc., Chicago, IL, USA), R software v3.6.3 (http://www.r-projiect.org/), and Prism 8.0 (GraphPad Software, Inc). Data were considered significant at *P* < 0.05.

## Results

The present study explored the effects of eight E2Fs on the clinical characteristics of LGG and their potential functions based on the CGGA, TCGA, and GSE16011 datasets. We then employed the LASSO Cox regression algorithm to develop a potential risk score. Two categories of patients with LGG were identified based on the median risk scores. A nomogram was formulated based on the results of the multivariate analysis. RT-qPCR and immunohistochemical analyses were used to validate the bioinformatics results.

### Relationships between E2Fs expression and the clinical characteristics of patients with LGG

**Figure 1.**
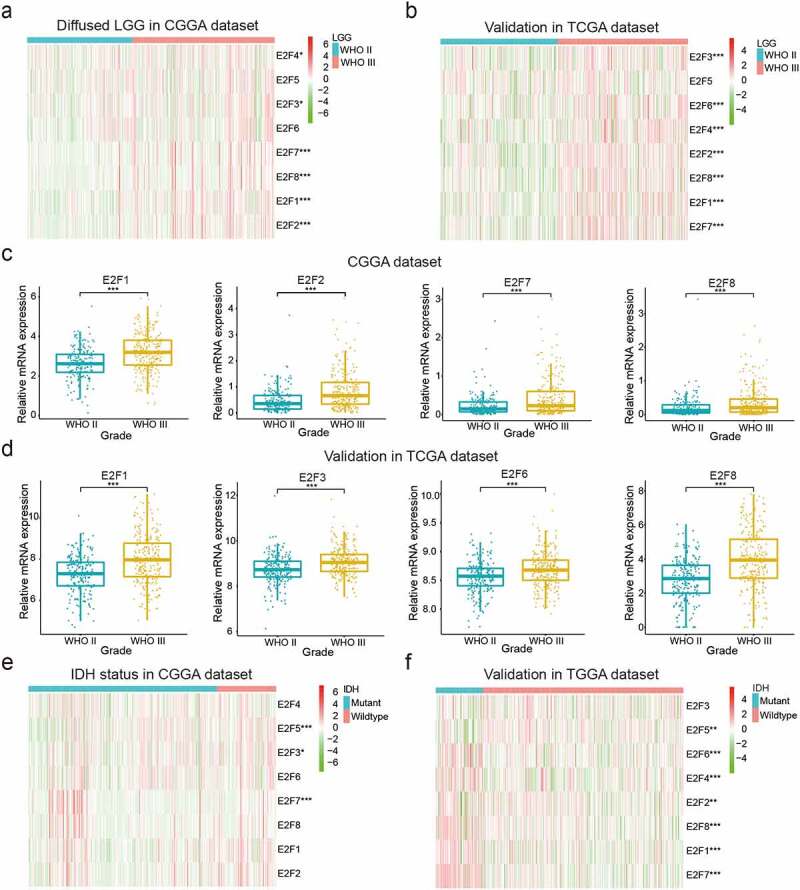
Correlations between the expression levels of E2F members and different clinical characteristics of patients with LGG. (a-d): Expression of eight E2F genes in LGG of different WHO grades. (e-f): Expression of eight E2F genes in patients with differential IDH status from the CGGA (e) and TCGA datasets (f). * *P*< 0.05, ** *P*< 0.01, and *** *P*< 0.001

Accumulating evidence suggests that E2Fs are significantly associated with apoptosis, metabolism, and angiogenesis in several tumor types [14–18]. To explore the potential involvement of E2F members in the malignancy of LGG, we systematically analyzed the associations between the expression patterns of the eight E2Fs and different clinicopathological features. To this end, we examined 443 patients with LGG from the CGGA dataset as the training set, 462 and 109 patients with LGG from the TCGA and GSE16011 datasets, respectively, as the validation set (Supplementary Table 1). A heatmap was generated to explore the associations between E2Fs expression and WHO grades in the training (CGGA) and validation (TCGA and GSE16011) datasets (Figure 1(a-b); Supplementary Figure 1(a)). Most E2Fs were critically associated with WHO grade. Our results showed marked upregulation of E2F1, E2F2, E2F3, E2F6, E2F7, and E2F8 in WHO III glioma (Figure 1(c-d); Supplementary Figure 1(b)). We further explored the correlations between E2Fs expression and isocitrate dehydrogenase (IDH) status in the training (CGGA) and validation (TCGA) datasets (Figure 1(e-f)), which revealed that wildtype IDH status was positively correlated with E2F3, E3F5, and E2F7 expression in the CGGA dataset, and with E2F1, E2F2, E2F4, E2F5, E2F6, E2F7, and E2F8 expression in the TCGA dataset. The difference in the correlation of wildtype IDH status and E2F expression profile between the two databases may be due to the many samples with missing IDH status in the CGGA dataset (Supplementary Table 1).

To explore whether E2Fs family members could serve as biomarkers for predicting the prognoses of LGG, we compared the transcript levels of the eight E2Fs between brain glioma and normal brain tissue specimens based on the ONCOMINE dataset. E2F5, E2F7, and E2F8 were significantly upregulated in brain and CNS cancers relative to normal brain samples, while E2F1 and E2F3 were more highly expressed in normal brain samples in the ONCOMINE dataset (Supplementary Figure 2a). We also explored the mutation rates of the eight E2Fs based on the TCGA and found very low rates for all E2Fs, indicating that the different expression levels of the eight E2Fs were not caused by genetic alterations (Supplementary Figure 2(b)). We then investigated the relationships between the eight E2Fs and tumor immunology and found that E2F1, E2F3, E2F5, E2F6, and E2F7 expression was positively correlated with many immune cells, which might explain their favorable prognostic value (Supplementary Figure 2(c)).

**Figure 2.**
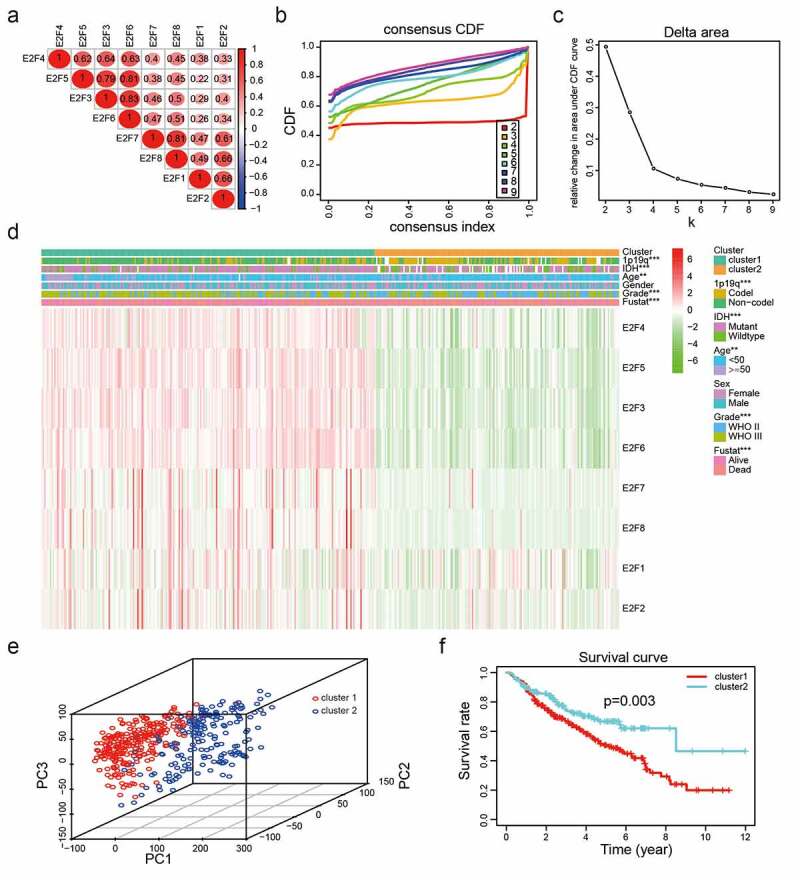
Two categories of patients based on distinct clinical characteristics and OS according to the gene expression of eight E2Fs in the CGGA dataset. (a): Spearman’s correlation analysis of the E2F family members. (b): CDFs for K = 2–9. (c): Delta areas under the CDF curves for K = 2–9. (d): Clinical characteristics of the two clusters defined based on the consensus expression of the eight members of the E2F family. (e): PCA of total RNA expression profile in the CGGA datasets. (f): Kaplan-Meier curves for samples in the CGGA datasets

### Categories based on consensus clustering of E2Fs are significantly correlated with LGG malignancy

To explore the interactions of the eight E2Fs, we analyzed their correlations according to their mRNA expression levels in the CGGA dataset. The expression levels of E2F1, E2F2, E2F3, E3F4, E2F5, E2F6, E2F7, and E2F8 were positively correlated with LGG (Figure 2(a)). Meanwhile, the PPI network of eight E2Fs constructed using STRING and Cytoscape showed that the E2F1 was a hub gene in the interactions of the eight E2Fs (Supplementary Figure 1(c)), indicating that E2F family members are linked to the promotion of malignant progression in LGG. The application of consensus clustering analysis led to the classification of patients into two categories based on the expression patterns of the eight E2F family members in the CGGA dataset (n = 443). A K value of 2 from among K values of 2–9 was selected using cumulative distribution function (CDF) curves and SigClust analysis (Figure 2(b-c)). The consensus score matrix of the expression levels of the eight E2Fs based on the CGGA dataset was generated to evaluate the influence of correlations within and between groups (Supplementary Figure 3), which led to the identification of two categories according to the different clinical characteristics of patients with LGG (Figure 2(d)). E2Fs transcript levels differed significantly between the two categories, as determined by PCA (Figure 2(e)). Furthermore, Kaplan-Meier analysis disclosed that compared to cluster2, the patients in cluster1 had poorer OS (*P*< 0.001; Figure 2(f)), based on the high levels of E2F1-8 in cluster1 (Supplementary Figure 4). In addition, the prevalence of high grade, old age, mutant-type IDH status, and 1p19q non-codeletion status were higher in cluster1 than those in cluster2 (Supplementary Table 3).

**Figure 3.**
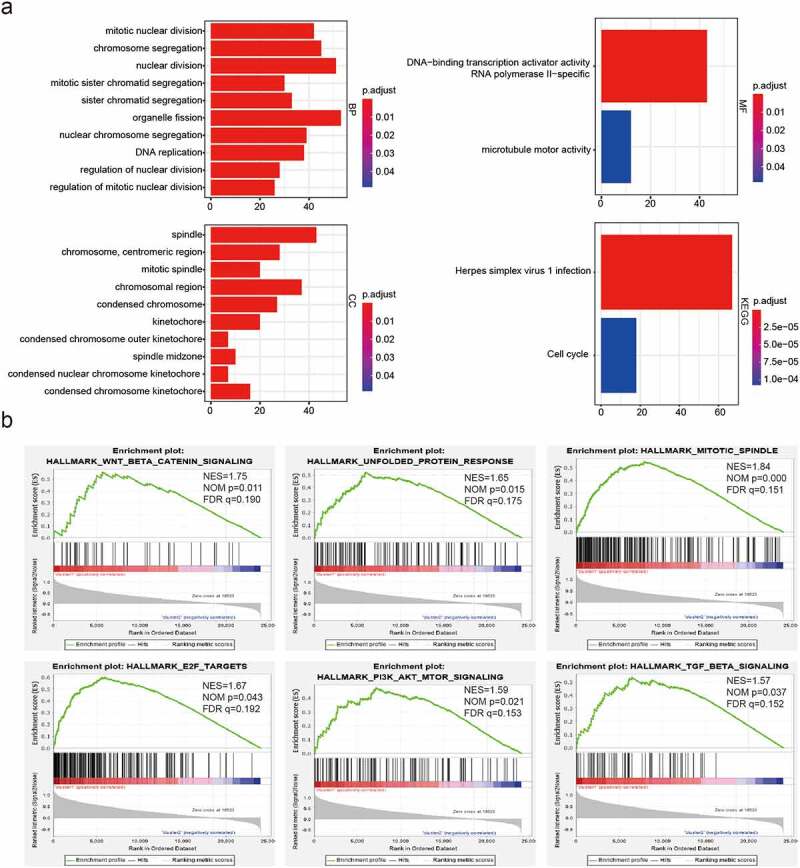
Functional annotations of differentially expressed genes between cluster1 and cluster2. (a): Functional annotations of differentially expressed genes via GO and KEGG pathway analyses. (b): Malignant hallmarks enriched in cluster1 determined using GSEA

**Figure 4.**
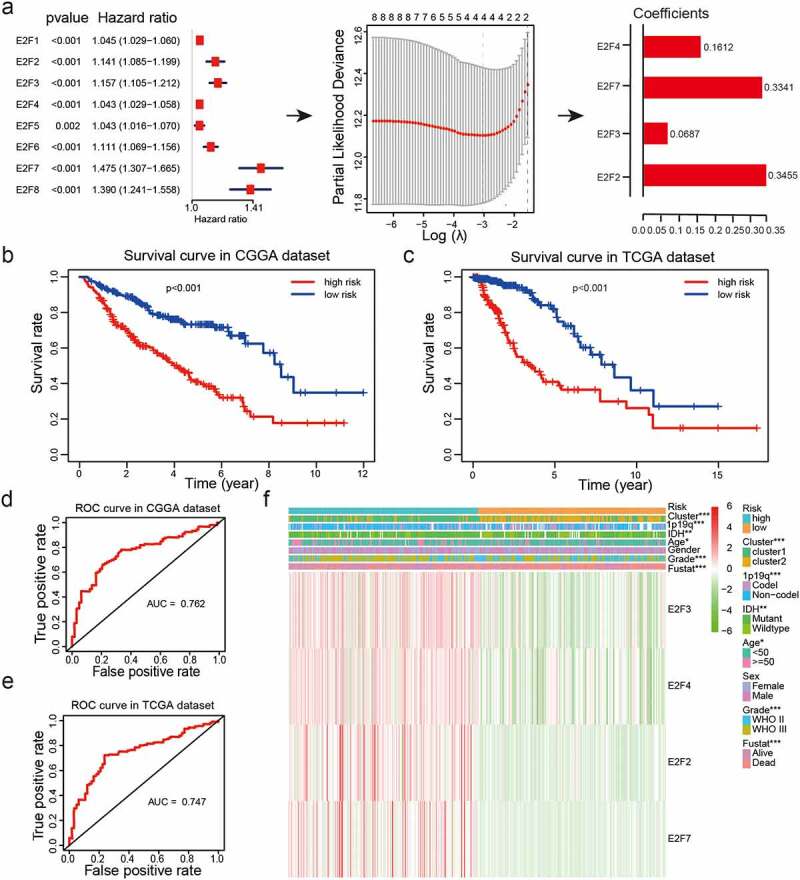
Risk models derived from expression patterns of four E2F genes. (a): The process of risk model construction. (b-c): Kaplan-Meier OS curves for patients from the CGGA (b) and TCGA (c) datasets categorized into two groups based on the median risk scores. (d-e): ROC curve analysis of the predictive efficiency of our risk model in the CGGA (d) and TCGA (e) datasets. (f): Heatmap of genes corresponding to four E2F genes and the distributions of clinical characteristics in the two subgroups. * *P*< 0.05, ** *P*< 0.01, and *** *P*< 0.001

### Molecular mechanisms by which E2F family members contribute to malignant progression in LGG

We identified genes that were significantly differentially expressed between cluster1 and cluster2 and further analyzed the malignancy-related biological processes enriched in cluster1 associated with these highly expressed genes were. We observed enrichment of genes involved in mitotic nuclear division, nuclear division, organelle fission, DNA replication, regulation of nuclear division, chromosomal region, spindle and microtubule motor activity (Figure 3a). KEGG pathway analysis further revealed a significant enrichment of genes involved in herpes simplex virus 1 infection and the cell cycle (Figure 3(a)). We also applied GSEA to identify the hallmarks of malignant tumors. Our results showed that Wnt beta-catenin signaling (NES = 1.75; normalized *P*= 0.011), mitotic spindle (NES = 1.84; normalized *P*< 0.001), unfolded protein response (NES = 1.65; normalized *P*< 0.05), and P13 K/Akt signaling pathway (NES = 1.59; normalized *P*< 0.05) were markedly correlated with cluster1 (Figure 3(b)). Thus, cluster1, as identified from consensus clustering, was a positively associated with malignancy progression in LGG.

### Prognostic value of E2Fs and a risk model derived from four selected E2F members

We explored the potential correlations between the eight E2Fs and prognosis using univariate Cox regression analysis of samples from the CGGA dataset. Expression of the eight E2Fs was strongly correlated with OS (*P*< 0.01) and all eight E2Fs were identified as high-risk genes (hazard ratio > 1; Figure 4(a)). Next, LASSO Cox regression analysis was performed using the CGGA dataset as a training set to construct an improved prognostic risk model according to E2Fs expression patterns (Figure 4(a)). Four genes were selected based on the minimum criteria to establish the risk score and the coefficients derived from the LASSO Cox regression analysis were used to calculate risk scores for all patients with LGG in both the training (CGGA) and validation (TCGA and GSE16011) datasets. The samples were categorized into low-risk and high-risk subgroups based on median risk scores from both datasets (Supplementary Table 4). Kaplan-Meier survival curves revealed a significant difference in OS between the low-risk and high-risk categories in both the training (CGGA) and validation (TCGA and GSE16011) datasets (*P*< 0.001; Figure 4b-c; Supplementary Figure 1(d)). We further evaluated the predictive value of our risk model with the aid of ROC curve analysis. The areas under the ROC curves (AUCs) for OS of patients was 0.762 in the CGGA dataset, 0.747 in the TCGA dataset, and 0.679 in the GSE16011 dataset, indicating that our risk model could accurately predict the OS of patients with LGG (Figure 4(d-e); Supplementary Figure 1(e)).

### The risk model has good prognostic performance and is significantly associated with the clinicopathological features of LGG

**Figure 5.**
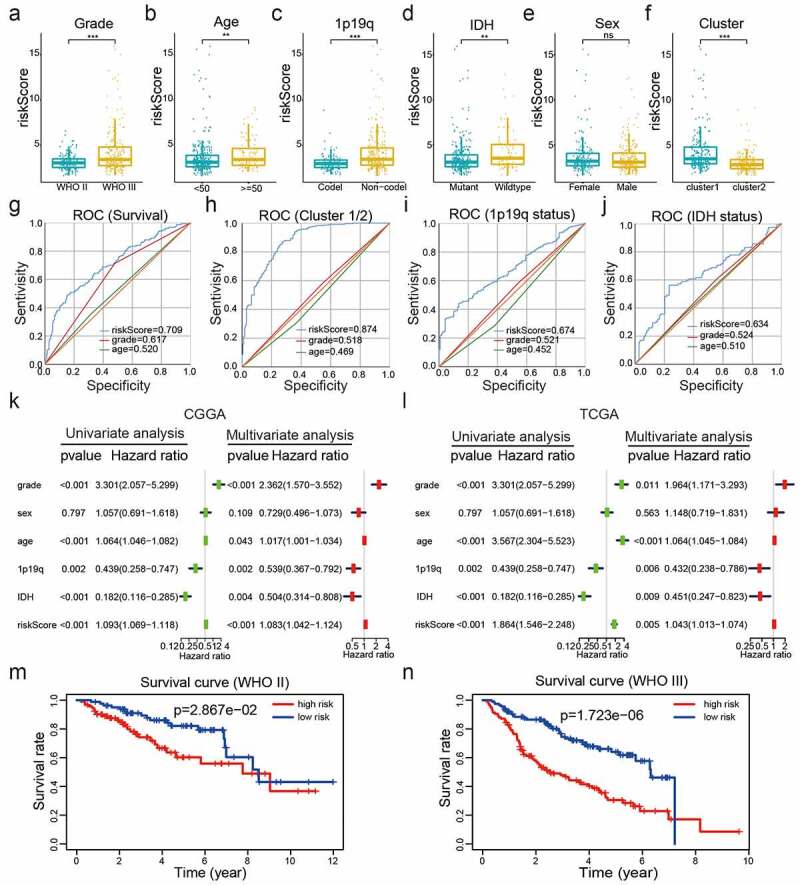
Correlations between risk scores, clinical characteristics and clusters. (a-f): Distributions of risk scores stratified by WHO grade (a), age (b), 1p/19q status (c), IDH status (d), sex (e), and cluster (f). (g-j): Predictive efficiency of risk score, WHO grade and age relative to the survival rate (g), cluster1 group (h), IDH status (i) and 1p/19q codel status (j). (k-l): Relationships between clinical characteristics and OS of patients in the CGGA (k) and TCGA (l) datasets determined via univariate and multivariate Cox regression analyses. (m-n): Kaplan-Meier analysis of gliomas different WHO grades from the CGGA dataset. ns *P*> 0.05, * *P*< 0.05, ** *P*< 0.01, and *** *P*< 0.001

Examination of the relationships between the risk scores and clinicopathological features of LGG from the CGGA datasets revealed significant differences between risk scores in groups stratified by cluster (*P*< 0.001), WHO grade (*P*< 0.001), age (*P*< 0.05), 1p/19q status (*P*< 0.001), survival status (*P*< 0.001), and IDH status (*P*< 0.01) (Figure 4(f); Supplementary Table 4; Figure 5(a-f)). Overall, our risk prognostic model accurately predicted the OS and clinical characteristics of patients with LGG. ROC curves were generated to validate the ability of our risk model to reliably predict the survival rate (AUC = 0.709), cluster1 category (AUC = 0.874), IDH mutant status (AUC = 0.674), and 1p19q codel status (AUC = 0.634; Figure 5(g-j)).

To establish whether the risk scores represented an independent prognostic index, we performed univariate and multivariate Cox regression analyses on both the training (CGGA) dataset and validation (TCGA and GSE16011) datasets. Univariate Cox regression analysis showed that WHO grade, age, IDH status, 1p/19q status, and risk score were strongly correlated with the OS of patients in the CGGA dataset. Multivariate Cox regression analysis revealed that WHO grade (*P*< 0.001), age (*P*< 0.05), IDH status (*P*< 0.01), 1p/19q status (*P*< 0.01), and risk score (*P*< 0.001) remained significantly correlated with OS (Figure 5(k)). Similar conclusions were reached from multivariate Cox regression analyses of the TCGA and GSE16011 datasets (Figure 5(l), Supplementary Figure 1(f)), which showed significant correlations between WHO grade (*P*< 0.05), age (*P*< 0.01),1p19q (*P*< 0.01), and risk scores (*P*< 0.01) and OS. Therefore, the risk scores derived from the four selected E2Fs genes showed strong prognostic value in LGG.

To explore the prognostic value of our risk model in LGG of different WHO grades, we analyzed the correlations between risk scores and OS via the Kaplan -Meier method, which revealed poor OS in the high-risk subgroups compared to those in the low-risk subgroups of different WHO grades from the CGGA dataset (Figure 5(m-n)). These collective findings indicated that our risk model had significant prognostic value for LGG of different WHO grades.

### Development of a nomogram based on clinicopathological features and risk score for predicting the prognosis of LGG

**Figure 6.**
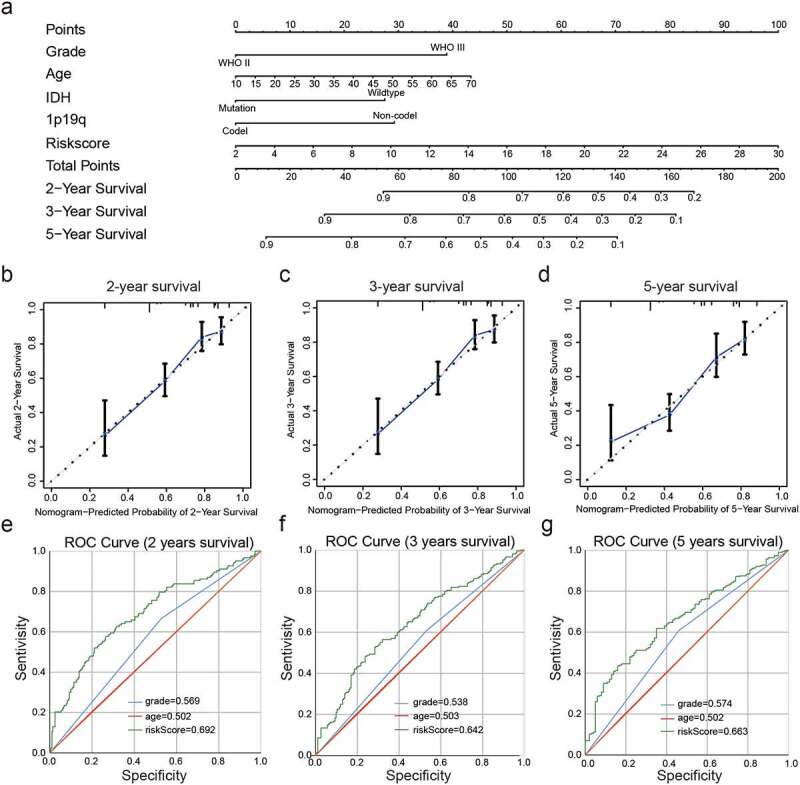
Construction and assessment of a nomogram to predict patient’ OS. (a): Nomogram based on the clinical characteristics and risk scores for predicting patient survival. (b-d): Calibration curve for predicting patient survival at 2 years (b), 3 years (c), and 5 years (d). (e-g): The predictive efficiency of the risk scores, WHO grade, and age showed by ROC curves based on 2- (e), 3- (f), and 5- (g) year survival rates

Then a nomogram was constructed for predicting LGG prognosis, integrating grade, age, IDH status, 1p19q status and risk scores based on the CGGA dataset (Figure 6(a)), and showed a C-index for survival prediction of 0.735.The calibration curve for the probabilities of survival at 2-, 3-, and 5- years showed optimal agreement between nomogram prediction and the actual observed outcomes (Figure 6(b-d)).The AUC for OS was 0.692 at 2 years, 0.642 at 3 years, and 0.663 at 5 years, showing the reliable predictive ability in the CGGA dataset (Figure 6(e-g)).

### The mRNA and protein expression of four selected E2F members in LGG

**Figure 7.**
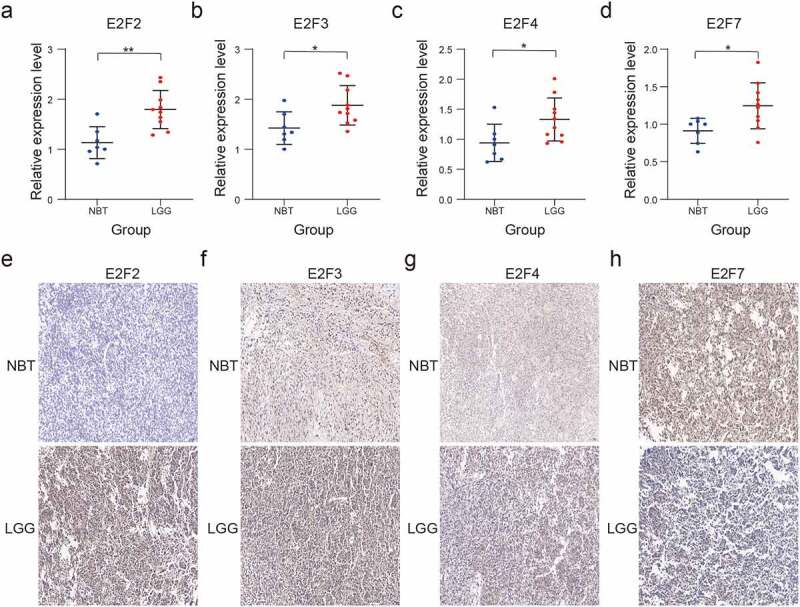
Validation of four selected E2F members by RT-qPCR and immunohistochemistry analysis. (a-d): Comparative E2F2 (a), E2F3 (b), E2F4 (c), and E2F7 (d) mRNA expression levels in NBT and LGG tissues. (e-h): Comparative E2F2 (e), E2F3 (f), E2F4 (g), and E2F7 (h) protein expression levels in NBT and LGG tissues by immunohistochemistry assay. ns *P* > 0.05, * *P* < 0.05, ** *P* < 0.01, and *** *P* < 0.001

We performed RT-qPCR and immunohistochemistry assays to assess the four E2F members to construct the risk scores. The RT-qPCR assay showed different mRNA expression level among the four E2F members (E2F2, E2F3, E2F4, and E2F7) between NBT and LGG (Figure 7(a-d)). The results of the immunohistochemistry assay also showed different protein expression levels of the four E2Fs between NBT and LGG tissue samples, consistent with the results of the bioinformatics analysis (Figure 7(e-h)).

## Discussion

Glioma is the most common lethal CNS tumor type in adults [29–31]. Precise surgical resection with preoperative imaging, adjuvant chemotherapy and postoperative radiotherapy constitute the standard treatment strategies for gliomas [32–35]. However, the OS rate of patients with glioma remains poor following standard therapy. LGG can evolve into high-grade gliomas and develop resistance to chemotherapy. Accumulating evidence suggests that E2Fs have significant prognostic value in multiple cancer types and may serve as potential diagnostic biomarkers [4,16–18].

Among the eight E2F family members, up-regulated E2F1 expression gene reportedly induced apoptosis in glioma cell lines [36,37], while E2F3 knockdown inhibited proliferation and migration in glioma [38]. Yu et al. investigated the expression of E2Fs and their prognostic value in high-grade glioma based on the TCGA dataset and identified E2F7 and E2F8 as novel potential prognostic markers linked with aggressive oncogenic processes [39]. Yin et al. also showed that E2F7 expression was significantly associated with poor prognosis in patients with gliomas [40]. However, conflicting data suggesting that atypical repressor E2Fs may have tumor suppressive and oncogenic functions in different tumor types have been reported [41–44]. For instance, E2F8 knockdown inhibited tumor formation in xenograft models of hepatocellular carcinoma and lung cancer in one study but significantly suppressed stress-induced skin cancer in another study using knockout mice [41]. E2F3B, an isoform of E2F3, usually acts as a canonical repressor but has also been reported as an oncogene in some tumor types [42–44].

Some studies have explored the mechanism of E2F transcription factors in the malignancy of glioma. Manzano et al. demonstrated that E2F1 modulated the expression of the anti-apoptotic molecule Bcl-2 and suggested that up-regulation of Bcl-2 might favor the oncogenic role of E2F1 and the other E2F family members in human glioma cells [45]. Zhang et al. reported that E2F2 enhanced PFKFB4 expression and regulated P13K/AKT phosphorylation to promote glioma malignancy [46].

In the present study, we evaluated the associations between the expression levels of eight E2F family members and the clinical characteristics of patients with LGG in training (CGGA) and validation (TCGA and GSE16011) datasets. As expected, most E2Fs (E2F1, E2F2, E2F3, E2F4, E2F7, and E2F8) were strongly associated with increasing LGG WHO grade, indicating that E2F gene expression is closely related to the clinical characteristics of malignancy. Our data based on samples from the ONCOMINE dataset, and the correlation with tumor immunology further support the utility of these eight E2F genes as biomarkers for predicting the malignancy of LGG.

We used consensus clustering analysis to sort patients with LGG into two clusters and found that cluster1 was strongly positively associated with poor OS, WHO grade III, IDH wild-type, older age, and 1p/19q non-codeletion of LGG. Multiple biological processes and signaling pathways involving E2F family members have been identified, including cell cycle [47,48], DNA repair [49], DNA replication [50,51], apoptosis [52], and p53 signaling [53]. In our study, E2Fs were associated with critical processes in LGG, including DNA replication, nuclear division, organelle fission, mitotic spindle, kinetochore, spindle midzone, and DNA-binding transcription activator, as well as signaling pathways underlying malignancy progression such as herpes simplex virus 1 infection and the cell cycle. Furthermore, GSEA revealed associations with several hallmarks of malignant tumors such as P13K-AKT-mTOR signaling, TGF-beta signaling, and unfolded protein response.

To ascertain whether E2F expression can be utilized as an independent prognostic index for the diagnosis of LGG, we derived a prognostic model using four selected E2Fs. Our results showed that a poor OS was strongly associated with the high-risk subgroup in both the training (CGGA) and validation (TCGA and GSE16011) datasets. We also observed a significant correlation between the risk scores of our prognostic model and the clinical characteristics of patients with LGG. Furthermore, our risk scores could serve as an independent prognostic index for the diagnosis of LGG, as determined by univariate and multivariate Cox regression analyses of both training (CGGA) and validation (TCGA and GSE16011) datasets. The risk scores based on the four selected E2Fs showed prognostic value for WHO grade II and III LGG. In addition, we constructed a nomogram to predict the prognosis of LGG, which integrated grade, age, IDH status, 1p19q status, and risk scores. The C-index for survival prediction and the AUC for OS showed the reliable ability of our nomogram in the CGGA dataset. Our results further indicated that the risk scores had clinical prognostic value in LGG.

## Conclusion

The expression levels of E2Fs were correlated with malignant clinical characteristics and cancer-related biological processes in LGG. The risk signature of the four selected E2Fs was an independent prognostic index for the diagnosis of LGG. Our results provide significant insights that offer a platform for further in-depth exploration of the specific roles of individual E2Fs in LGG.

## Supplementary Material

Supplemental Material

## Data Availability

Data for this work were obtained from the CGGA (http://www.cgga.org.cn/), TCGA (https://portal.gdc.cancer.gov/), GSE16011 (https://www.ncbi.nlm.nih.gov/geo/query/acc.cgi?acc=GSE16011), ONCOMINE (https://www.oncomine.org/), and Human Protein Atlas datasets (https://www.proteinatlas.org/).

## Highlights

1. The correlations between E2Fs expression and LGG malignancy were explored.

2. A risk signature of four E2Fs was an independent prognostic index for LGG prognosis.

3. The bioinformatics results were validated by RT-qPCR and immunohistochemistry assays.
