# Supplementary material for: Early 2 factor (E2F) transcription factors contribute to malignant progression and have clinical prognostic value in lower-grade glioma
Source: Bioengineered. 2021 Oct 7;12(1):7765–79. doi: 10.1080/21655979.2021.1985340 (PMC8806968; doi:10.1080/21655979.2021.1985340)
Supplement: Supplemental Material [file KBIE_A_1985340_SM1410.zip › supplementary/Supplementary Fig legends.docx]

**Supplementary file 1 Figure 1.** The results of our bioinformatics analysis were validated using the GSE16011 datasets. (a-b): Expression of eight E2F genes in LGG of different WHO grades. (c): The PPI network demonstrating E2F1 was a hub gene in the interactions of the eight E2Fs. (d): Kaplan-Meier OS curves of patients from the GSE16011 datasets. (e): ROC curve analysis of the predictive efficiency of our risk model in the GSE16011 dataset. (f): Relationships between clinical characteristics and OS of patients in the GSE16011 dataset determined via univariate and multivariate Cox regression analyses.

**Supplementary file 2 Figure 2.** Landscape of genetic and expression variation of eight E2F members in glioma. (a) The numbers of datasets with statistically significant mRNA over-expression (red) or downregulated expression (blue) in brain and central nervous system cancer samples from the ONCOMINE dataset. (b) The mutation frequencies of eight E2F members in the TCGA dataset. The upper and lower bar plots indicate the proportion of each variant type and the sample annotations, respectively. (c) Correlation heatmap between eight E2F members and immune cells in the CGGA dataset. Orange and blue indicate positive and negative correlations, respectively.

**Supplementary file 3 Figure 3.** Consensus score matrix of the eight E2F family members in the LGG from the CGGA dataset. Consensus score matrices for K = 2 (a), K = 3 (b), K = 4 (c), and K = 5 (d).

**Supplementary file 4 Figure 4.** Differences in the expression patterns of the eight E2Fs between two LGG clusters based on the consensus clustering analysis. Comparisons of E2F1 (a), E2F2 (b), E2F3 (c), E2F4 (d), E2F5 (e), E2F6 (f), E2F7 (g), and E2F8 (h) expression levels between cluster1 and cluster2.

**Supplementary file 5 Table 1.** Clinicopathological information for the CGGA, TCGA, and GSE16011 datasets.

**Supplementary file 6 Table 2.** Primer sequences used for RT-qPCR analysis.

**Supplementary file 7 Table 3.** Clinicopathological features of the clusters included in this study.

**Supplementary file 8 Table 4.** Differences in clinicopathological features between the low-risk and high-risk subgroups.
