# Supplementary material for: Early 2 factor (E2F) transcription factors contribute to malignant progression and have clinical prognostic value in lower-grade glioma
Source: Bioengineered. 2021 Oct 7;12(1):7765–79. doi: 10.1080/21655979.2021.1985340 (PMC8806968; doi:10.1080/21655979.2021.1985340)
Supplement: Supplemental Material [file KBIE_A_1985340_SM1410.zip › supplementary/Table S1.docx]

**Table S1.** The clinicopathological information for the CGGA, TCGA and GSE16011 datasets

|  | | CCGA dataset | | TGGA dataset | | GSE16011 dataset | |
| --- | --- | --- | --- | --- | --- | --- | --- |
|  |  | Number | Percentage | Number | Percentage | Number | Percentage |
| Total |  | 443 | 100.00% | 462 | 100.00% | 109 | 100.00% |
| Age |  | 11-72 (40) |  | 14-87 (41) |  | 23-81 |  |
|  | <median | 205 | 46.28% | 241 | 52.16% | 55 | 50.46% |
|  | ≥median | 238 | 53.72% | 221 | 47.83% | 54 | 49.54% |
| Sex |  |  |  |  |  |  |  |
|  | Female | 192 | 43.34% | 207 | 44.80% | 37 | 33.94% |
|  | Male | 251 | 56.66% | 255 | 55.19% | 72 | 66.06% |
| Grade |  |  |  |  |  |  |  |
|  | II | 188 | 42.44% | 219 | 47.40% | 24 | 22.01% |
|  | III | 255 | 57.56% | 243 | 52.59% | 85 | 77.98% |
| IDH |  |  |  |  |  |  |  |
|  | Mutation | 306 | 69.07% | 373 | 80.74% | 22 | 20.18% |
|  | Wildtype | 96 | 21.67% | 87 | 18.83% | 41 | 37.61% |
|  | NA | 41 | 9.26% | 2 | 0.43% | 46 | 42.20% |
| 1p19q |  |  |  |  |  |  |  |
|  | Codel | 131 | 29.57% | 152 | 32.90% | 43 | 39.45% |
|  | Non-codel | 273 | 61.63% | 310 | 67.10% | 29 | 26.66% |
|  | NA | 39 | 8.80% | 0 | 0.00% | 37 | 33.94% |
