# Supplementary material for: Early 2 factor (E2F) transcription factors contribute to malignant progression and have clinical prognostic value in lower-grade glioma
Source: Bioengineered. 2021 Oct 7;12(1):7765–79. doi: 10.1080/21655979.2021.1985340 (PMC8806968; doi:10.1080/21655979.2021.1985340)
Supplement: Supplemental Material [file KBIE_A_1985340_SM1410.zip › supplementary/Table S2.docx]

**Table S2.** Primer sequences used for Realtime PCR analysis

| **Gene symbol** | **Gen Bank Accession no.** | **Primer set sequence (5’->3’)** | **Amplicon size (bp)** |
| --- | --- | --- | --- |
| *E2F2* | NM_004091.4 | Forward:  CAACATCCAGTGGGTAGGCA  Reverse:  GGCAATCACTGTCTGCTCCT | 241 |
| *E2F3* | NM_001949.5 | Forward:  GCGGTATGATACGTCTCTTGGT  Reverse:  AGACTGCAGCCCATCCATTG | 207 |
| *E2F4* | NM_001950.4 | Forward:  GATCCCACACGAGAGTGCAT  Reverse:  AAGCAGAGGGGCAAACACTT | 75 |
| *E2F7* | NM_203394.3 | Forward:  TAGGAAAGCAGGGATGGAGG  Reverse: TATTGGAGTCTTCGGGGCCA | 154 |
