# Supplementary material for: Early 2 factor (E2F) transcription factors contribute to malignant progression and have clinical prognostic value in lower-grade glioma
Source: Bioengineered. 2021 Oct 7;12(1):7765–79. doi: 10.1080/21655979.2021.1985340 (PMC8806968; doi:10.1080/21655979.2021.1985340)
Supplement: Supplemental Material [file KBIE_A_1985340_SM1410.zip › supplementary/Table S3.docx]

**Table S3.** Clinicopathological features of the clusters included in this study

| CCGA dataset | | | | |
| --- | --- | --- | --- | --- |
|  |  | Cluster 1 | Cluster 2 | *P*-value |
| Total cases |  | 233 | 171 |  |
| Sex |  |  |  | 0.08 |
|  | Male | 133 | 96 |  |
|  | Female | 100 | 75 |  |
| Age |  |  |  | <0.05 |
|  | <50 | 40 | 34 |  |
|  | >=50 | 193 | 137 |  |
| Grade |  |  |  | <0.001 |
|  | II | 96 | 77 |  |
|  | III | 137 | 94 |  |
| IDH |  |  |  | <0.001 |
|  | Mutation | 187 | 89 |  |
|  | Wildtype | 43 | 48 |  |
|  | NA | 3 | 34 |  |
| 1p19q |  |  |  | <0.001 |
|  | Codel | 71 | 47 |  |
|  | Non-codel | 160 | 89 |  |
|  | NA | 2 | 35 |  |
| Survival state |  |  |  | <0.001 |
|  | Alive | 126 | 121 |  |
|  | Dead | 107 | 50 |  |
