# Supplementary material for: Early 2 factor (E2F) transcription factors contribute to malignant progression and have clinical prognostic value in lower-grade glioma
Source: Bioengineered. 2021 Oct 7;12(1):7765–79. doi: 10.1080/21655979.2021.1985340 (PMC8806968; doi:10.1080/21655979.2021.1985340)
Supplement: Supplemental Material [file KBIE_A_1985340_SM1410.zip › supplementary/Table S4.docx]

**Table S4.** Clinicopathological features are different between low-risk and high-risk

| CGGA dataset | | | | |
| --- | --- | --- | --- | --- |
|  |  | low-Risk | high-Risk | P-value |
| Total cases |  | 202 | 202 |  |
| Cluster |  |  |  | <0.001 |
|  | Cluster 1 | 62 | 171 |  |
|  | Cluster 2 | 140 | 31 |  |
| Sex |  |  |  | <0.05 |
|  | Male | 118 | 111 |  |
|  | Female | 84 | 91 |  |
| Age |  |  |  | <0.001 |
|  | ＜50 | 165 | 165 |  |
|  | >=50 | 37 | 37 |  |
| Grade |  |  |  |  |
|  | II | 107 | 66 | <0.001 |
|  | III | 95 | 136 |  |
| IDH |  |  |  | <0.001 |
|  | Mutation | 132 | 144 |  |
|  | Wildtype | 43 | 48 |  |
|  | NA | 27 | 10 |  |
| 1p19q |  |  |  | <0.001 |
|  | Codel | 71 | 47 |  |
|  | Non-codel | 101 | 148 |  |
|  | NA | 30 | 7 |  |
| Survival state |  |  |  |  |
|  | Alive | 107 | 66 | <0.001 |
|  | Dead | 95 | 136 |  |
