# Supplementary figures and images for: Early 2 factor (E2F) transcription factors contribute to malignant progression and have clinical prognostic value in lower-grade glioma
Source: Bioengineered. 2021 Oct 7;12(1):7765–79. doi: 10.1080/21655979.2021.1985340 (PMC8806968; doi:10.1080/21655979.2021.1985340)

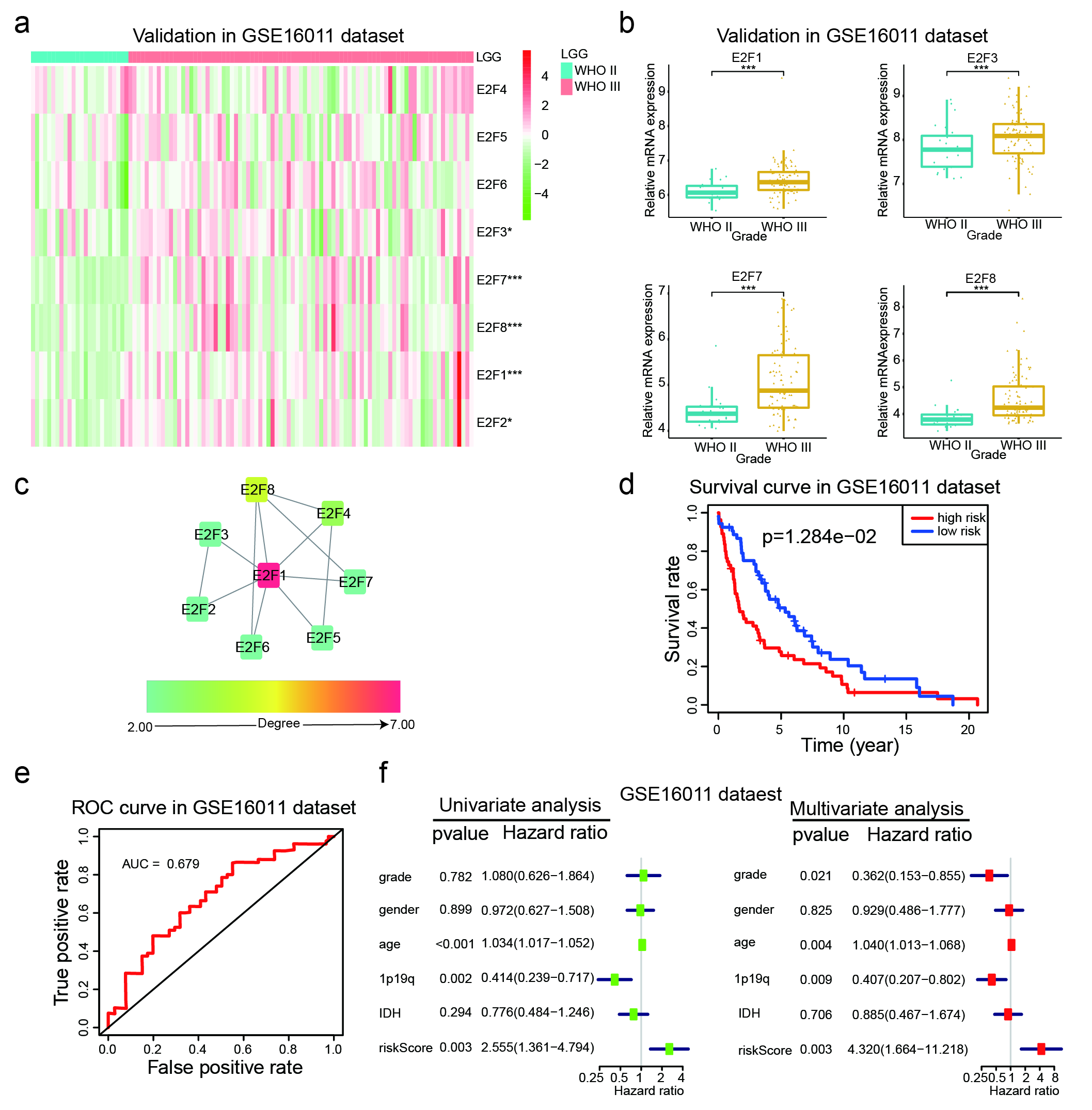

Supplement: Supplemental Material [file KBIE_A_1985340_SM1410.zip › supplementary/Figure S1.tif]

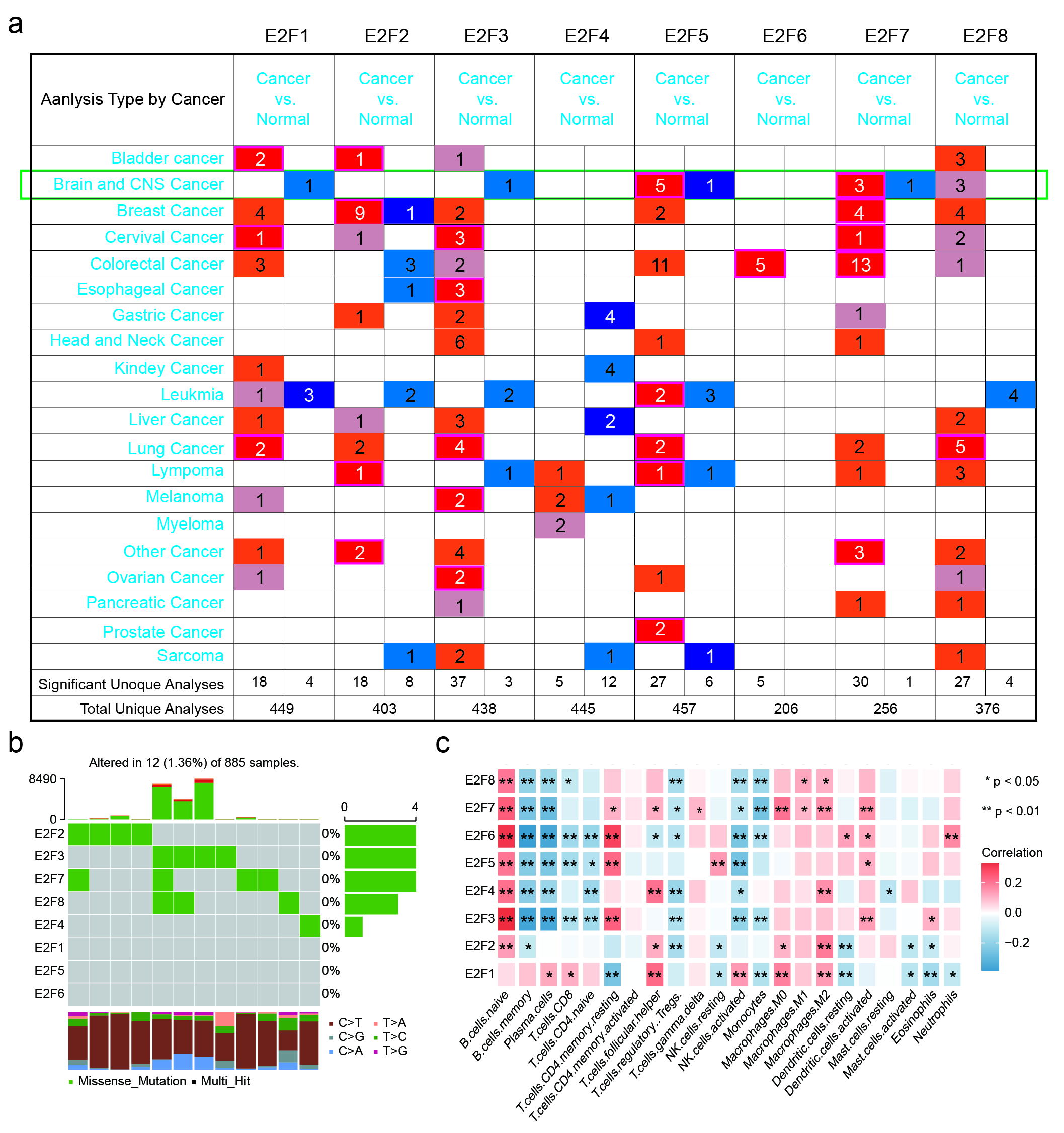

Supplement: Supplemental Material [file KBIE_A_1985340_SM1410.zip › supplementary/Figure S2.tif]

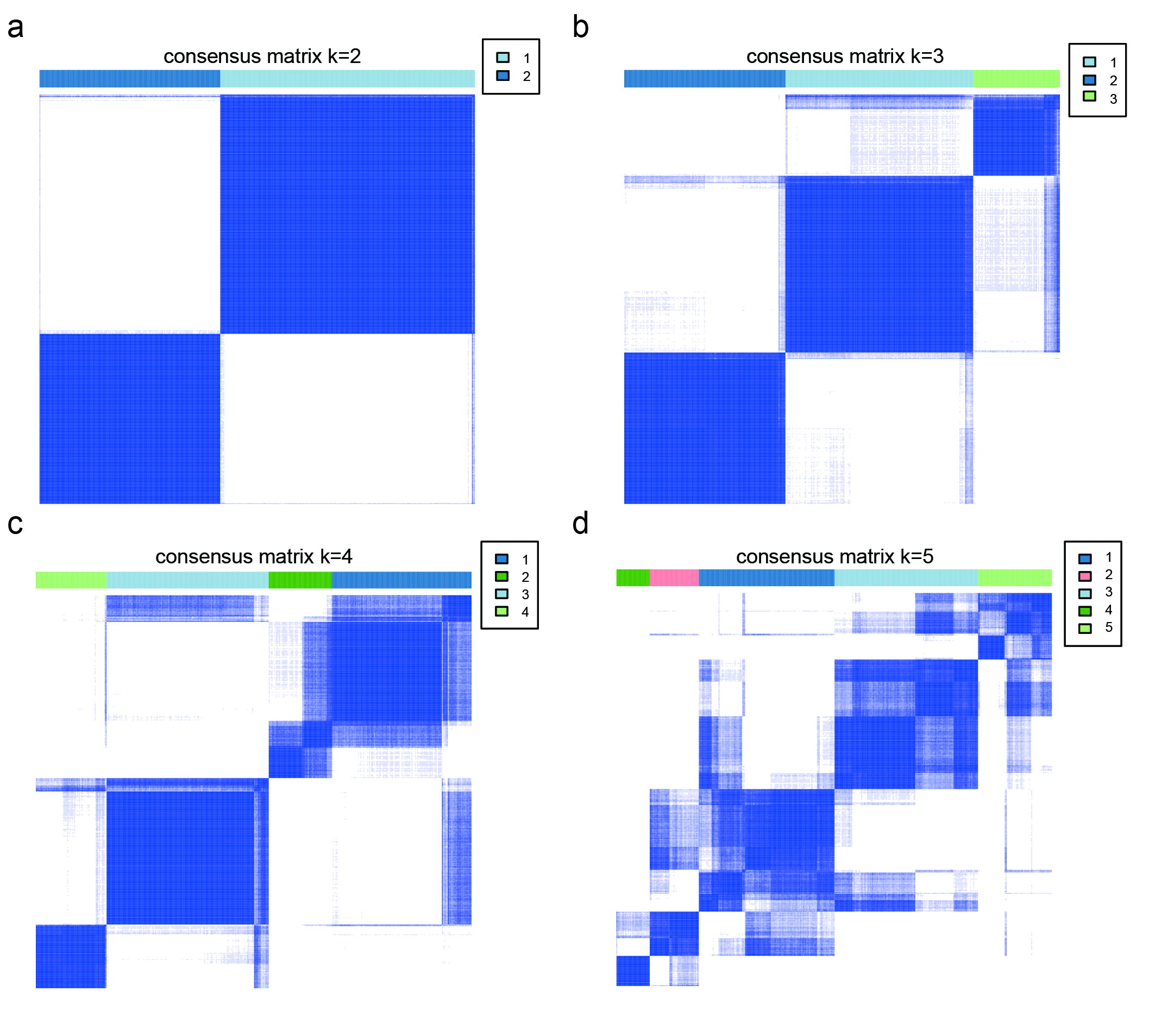

Supplement: Supplemental Material [file KBIE_A_1985340_SM1410.zip › supplementary/Figure S3.tif]

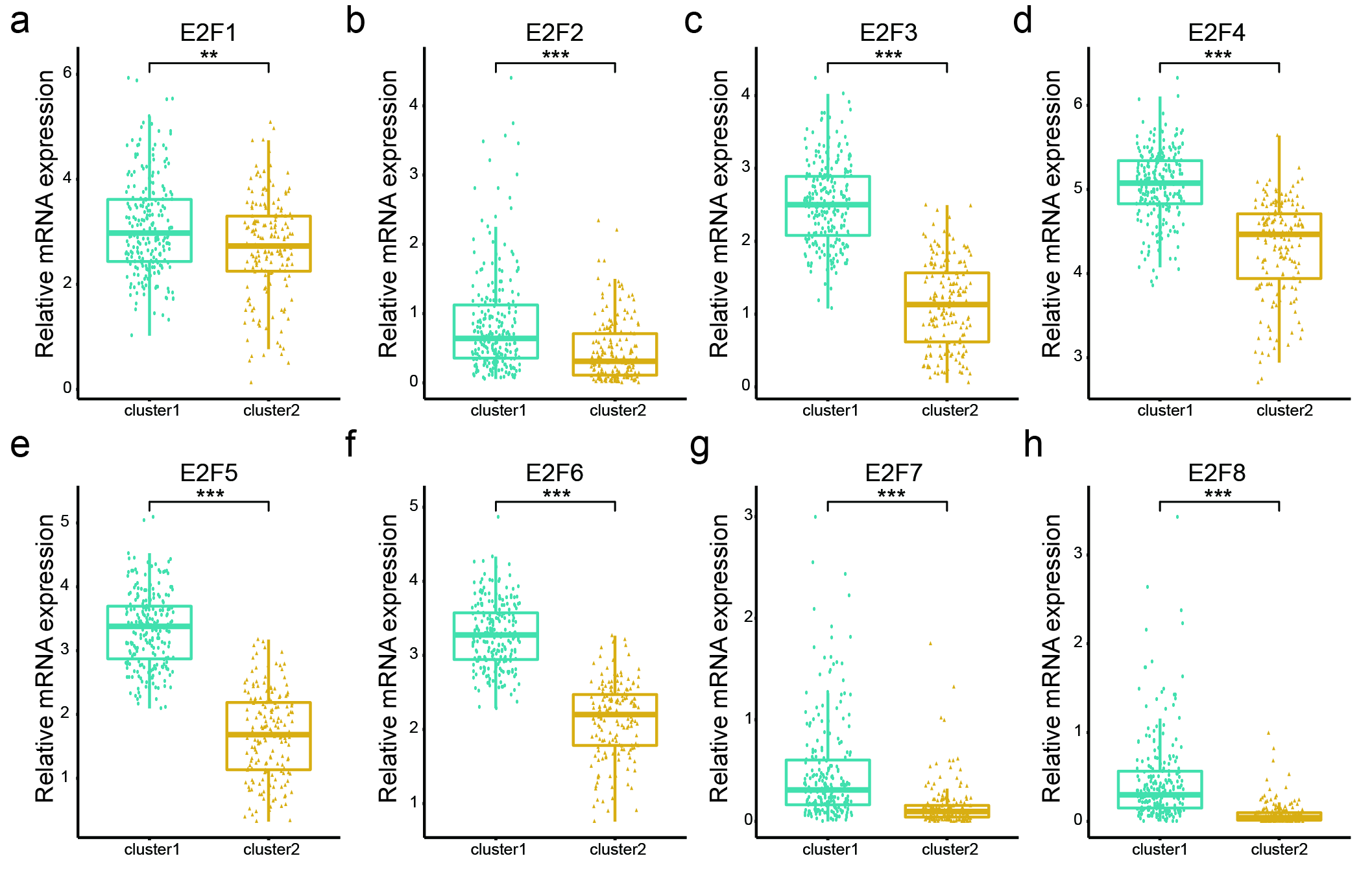

Supplement: Supplemental Material [file KBIE_A_1985340_SM1410.zip › supplementary/Figure S4.tif]
